# Early Evaluation of the Food and Drug Administration (FDA) Adverse Event Monitoring System (AEMS): An Analysis of Over 32 Million Pharmacovigilance Reports

**DOI:** 10.7759/cureus.106259

**Published:** 2026-04-01

**Authors:** Shaheen E Lakhan

**Affiliations:** 1 Bioscience, Global Neuroscience Initiative Foundation, Miami, USA; 2 Bioscience, Boricua Bio, San Juan, USA; 3 Neurology, Western University of Health Sciences, Pomona, USA; 4 Neurology, A.T. Still University School of Osteopathic Medicine in Arizona, Mesa, USA; 5 Medicine, Morehouse School of Medicine, Atlanta, USA

**Keywords:** adverse events, drug safety, faers, fda, pharmacovigilance

## Abstract

Introduction: Post-marketing pharmacovigilance systems are essential for detecting adverse drug reactions not identified during pre-marketing clinical trials. The United States Food and Drug Administration (FDA) introduced the Adverse Event Monitoring System (AEMS) public dashboard on March 11, 2026, a new platform that provides near-real-time access to data from the FDA Adverse Event Reporting System (FAERS). This interface allows clinicians and researchers to query pharmacovigilance data through an interactive visualization platform without requiring advanced data processing. To describe the structure, available features, and reporting patterns of the newly released AEMS dashboard, this study provides an exploratory descriptive analysis of FAERS report characteristics.

Methods: A cross-sectional descriptive analysis was conducted using data available through the FDA AEMS public dashboard on the day of its public release. The dashboard modules were explored, and datasets were exported using the built-in data download functionality. Variables examined included total report counts, seriousness of reports, death outcomes, patient sex distribution, reporter type, reporting region, annual reporting trends, and products with the highest total adverse event counts. Descriptive statistics were used to summarize available pharmacovigilance data.

Results: At the time of analysis, the AEMS dashboard contained 32,815,201 adverse event reports submitted to FAERS. Among these reports, 17,929,119 reports (54.6%) were classified as serious (excluding death), while 2,899,382 reports (8.8%) involved death outcomes. Female patients accounted for 17,292,788 (52.7%) reports, compared with 11,455,663 (34.9%) reports among male patients, while 4,066,750 (12.4%) reports did not specify sex. Annual report volumes increased substantially after 2014, exceeding two million reports annually during recent years, with 2,359,318 reports recorded in 2022 and 2,211,235 reports recorded in 2023.

Conclusions: This study provides one of the earliest evaluations of the newly released FDA AEMS dashboard and demonstrates its capability to support rapid exploratory pharmacovigilance analysis. The use of publicly accessible data improves transparency and reproducibility of pharmacovigilance research. However, inherent limitations of spontaneous reporting databases, including underreporting, reporting bias, incomplete clinical data, and inability to establish causality, remain important considerations when interpreting findings derived from the system.

## Introduction

Post-marketing drug safety surveillance plays a critical role in identifying adverse drug reactions that may not be detected during clinical trials [1]. Clinical trials are typically limited in size, duration, and population diversity, which may prevent the detection of rare or delayed adverse events [2]. Consequently, pharmacovigilance systems are essential for monitoring the safety of drugs and biologics after regulatory approval [2].

The United States Food and Drug Administration (FDA) Adverse Event Reporting System (FAERS) is one of the largest pharmacovigilance databases in the world and serves as the primary mechanism by which the FDA monitors post-marketing drug safety [3]. FAERS collects adverse event reports submitted by healthcare professionals, consumers, and pharmaceutical manufacturers. These reports are used to identify potential safety signals and support regulatory decision-making.

In addition to US-based pharmacovigilance systems, several large international adverse event reporting databases support global drug safety monitoring. These include VigiBase [4], maintained by the World Health Organization, which aggregates individual case safety reports from over 100 countries, and EudraVigilance [5], the European Union's centralized system for monitoring suspected adverse drug reactions. Together, these platforms complement FAERS by enabling the detection of safety signals across diverse populations and regulatory settings.

Historically, access to FAERS data required downloading quarterly data files and performing complex database processing, which limited use to researchers with specialized technical expertise and infrastructure. In contrast, the FDA recently introduced the Adverse Event Monitoring System (AEMS) public dashboard on March 11, 2026, a unified web-based platform that provides a near-real-time visualization interface layered on FAERS, enabling direct exploratory analysis [6]. While FAERS functions as the underlying data repository, AEMS represents an integrated visualization interface that allows users to directly explore adverse event reports across multiple FDA-regulated product categories. The system consolidates several legacy reporting databases into a single platform and provides tools to examine reports by drug product, reaction term, demographics, reporter type, geographic region, and reporting year.

The purpose of this study is to provide an early descriptive evaluation of the newly released AEMS dashboard, describe the data available through the system, and discuss its potential applications for pharmacovigilance research. The development of AEMS represents part of a broader FDA initiative to modernize post-marketing surveillance infrastructure and increase the transparency of adverse event reporting data [6]. 

## Materials and methods

Study design

A descriptive cross-sectional analysis was conducted using data obtained from the FDA AEMS public dashboard from database inception to March 11, 2026 [7]. The study did not involve human subjects research because all data were publicly available and fully de-identified. All analyses reflect default, unfiltered outputs from the AEMS dashboard as displayed on March 11, 2026, without the application of additional query parameters or user-defined filters.

Data source

Data were obtained from the publicly accessible AEMS dashboard maintained by the US FDA. The platform provides an interactive interface for querying data from the FAERS, which contains adverse event reports submitted by healthcare professionals, consumers, and pharmaceutical manufacturers. All data reflect records available in the AEMS dashboard at the time of access on March 11, 2026, and may change as additional FAERS reports are incorporated.

Data access

The AEMS dashboard was accessed on the day of its public release. Data were extracted using the dashboard's built-in export functionality. The dashboard provides near-real-time updates and allows users to download aggregated data tables for further analysis.

To evaluate product-level reporting patterns, additional analyses were conducted using the publicly available OpenFDA application programming interface (API) [8], which enables programmatic access to FAERS data. Because the AEMS dashboard does not provide native functionality to rank products by total adverse event report counts, product-level aggregation was performed externally. Adverse event reports were grouped by medicinal product name using the OpenFDA endpoint "patient.drug.medicinalproduct.exact", and products were ranked based on total report counts. Due to variability in drug naming conventions within FAERS, including differences in capitalization, punctuation, and brand versus generic naming, counts reflect exact string matches and may include multiple entries corresponding to the same active ingredient.

Variables examined

The following variables were analyzed: total number of FAERS reports, number of serious reports, number of death reports, patient sex distribution, reporter type, reporting region, annual reporting trends, and top 10 products by number of adverse event reports.

Data analysis

Descriptive statistics were used to summarize the available data. Report counts and proportions were calculated for demographic and outcome variables. Data were organized and analyzed using Microsoft Excel for Microsoft 365 (Microsoft Corporation, Redmond, Washington, United States). No patient-level identifiable information was accessed, and all data were publicly available in aggregated form. Analyses were conducted at the report level, as presented within the AEMS dashboard, rather than at the case level.

No additional deduplication of reports was performed beyond the processing inherent to the AEMS system, and potential duplicate reports within FAERS may influence aggregate counts. Missing data were retained and reported as "not specified" categories, with no imputation performed.

## Results

Overall FAERS report characteristics

A total of 32,815,201 adverse event reports were identified in the FAERS database as displayed through the AEMS dashboard [7] during the study period. Among these, 17,929,119 reports were classified as serious (excluding death), and 2,899,382 reports involved death outcomes. These reports represent adverse events associated with drugs and biologic products submitted by manufacturers, healthcare professionals, and consumers through the FAERS pharmacovigilance reporting system. Key characteristics of FAERS reports available through the AEMS dashboard are summarized in Table 1.

**Table 1:** Summary of FAERS reports available through the FDA AEMS dashboard Summary of adverse event reports available through the FDA AEMS dashboard at the time of analysis on March 11, 2026. FAERS: Food and Drug Administration Adverse Event Reporting System; FDA: Food and Drug Administration; AEMS: Adverse Event Monitoring System

Characteristic	Number of reports	Percentage (%)
Total reports	32,815,201	100
Serious reports (excluding death)	17,929,119	54.6
Death outcomes	2,899,382	8.8
Non-serious reports	11,986,700	36.5
Female patients	17,292,788	52.7
Male patients	11,455,663	34.9
Sex not specified	4,066,750	12.4
Healthcare professional reporters	16,018,871	48.8
Consumer/non-health professional reporters	15,765,319	48
Reporter type not specified	1,030,426	3.1
Domestic reports (United States)	22,412,950	68.3
Foreign reports	10,357,752	31.6
Region not specified	44,499	0.14

Distribution of reports by sex

Adverse event reports were more frequently reported among female patients. Female patients accounted for 17,292,788 reports (52.7%), while 11,455,663 reports (34.9%) involved male patients. In 4,066,750 reports (12.4%), patient sex was not specified. These findings indicate a higher representation of female patients in FAERS adverse event reporting. This pattern was consistently observed across multiple reporting years, with female reports exceeding male reports annually.

Temporal trends in adverse event reporting

Analysis of FAERS reports by received year demonstrated a sustained increase in adverse event reporting over time. Earlier years in the database contained comparatively fewer reports, while more recent years demonstrated substantially higher reporting volumes (Table 2). For example, 2,174,859 reports were recorded in 2019, increasing to 2,210,746 reports in 2020, 2,343,535 reports in 2021, and 2,359,318 reports in 2022. Reporting volumes remained elevated in subsequent years, with 2,211,235 reports recorded in 2023.

**Table 2:** Temporal trends in FAERS adverse event reporting by year Annual adverse event report counts in the FAERS, as visualized through the FDA AEMS dashboard, based on data accessed on March 11, 2026. Reporting volume increased substantially after 2014, exceeding two million reports annually in recent years. Lower counts in 2024-2026 reflect incomplete data due to reporting delays, ongoing case processing, and the partial reporting year for 2026. FAERS: Food and Drug Administration Adverse Event Reporting System; FDA: Food and Drug Administration; AEMS: Adverse Event Monitoring System

Year	Total reports
2014	1,198,411
2015	1,719,754
2016	1,683,291
2017	1,804,736
2018	2,139,631
2019	2,174,859
2020	2,210,746
2021	2,343,535
2022	2,359,318
2023	2,211,235
2024	2,108,659
2025	2,081,379
2026	386,791

Report counts for the most recent years (2024-2026) appear lower because FAERS data for recent reporting periods are incomplete at the time of analysis due to reporting delays, ongoing case processing, and the continuous addition of newly submitted reports to the database. In addition, 2026 represents a partial reporting year.

Reports by reporter type

Adverse event reports were submitted by several reporter categories. Healthcare professionals accounted for the largest proportion of submissions with 16,018,871 reports (48.8%), closely followed by consumer or non-health professional reporters with 15,765,319 reports (48%). Reports with unspecified reporter type accounted for 1,030,426 reports (3.1%), while a very small number of submissions were categorized as other reporter types (585 reports, <0.01%).

Unlike the traditional FAERS quarterly datasets in which manufacturer reports are categorized separately, the AEMS dashboard aggregates manufacturer submissions within the healthcare professional reporting category. As a result, the proportion of reports attributed to healthcare professionals in the AEMS interface likely reflects both direct clinician submissions and manufacturer-mandated safety reports.

Reports by age group

Adverse event reports were most frequently reported among adults aged 18-64 years, accounting for 11,509,558 reports (35.1%) (Table 3). Reports involving older adults aged 65-85 years accounted for 6,787,596 reports (20.7%), while 586,062 reports (1.8%) involved individuals older than 85 years. Pediatric reports were less frequent and included 84,879 reports in infants aged 0-1 month, 179,627 reports among children aged two months to two years, 476,534 reports among children aged 3-11 years, and 560,655 reports among adolescents aged 12-17 years. A substantial proportion of reports (12,630,290 reports, 38.5%) did not specify patient age.

**Table 3:** Distribution of FAERS adverse event reports by age group Distribution of adverse event reports by age group in the FDA AEMS dashboard, based on data accessed on March 11, 2026. Adults aged 18-64 years accounted for the largest proportion of reports, followed by older adults aged 65-85 years. A substantial proportion of reports did not include age information, reflecting incomplete data capture in spontaneous reporting systems. FAERS: Food and Drug Administration Adverse Event Reporting System; FDA: Food and Drug Administration; AEMS: Adverse Event Monitoring System

Age group	Number of reports	Percentage (%)
0-1 month	84,879	0.26
2 months-2 years	179,627	0.55
3-11 years	476,534	1.45
12-17 years	560,655	1.71
18-64 years	11,509,558	35.1
65-85 years	6,787,596	20.7
>85 years	586,062	1.8
Not specified	12,630,290	38.5

Geographic distribution of reports

Adverse event reports were most frequently submitted from the United States. Domestic reports accounted for 22,412,950 submissions (68.3%), while 10,357,752 reports (31.6%) originated from foreign sources. A small proportion of reports (44,499 reports, 0.14%) did not specify the geographic reporting region. The majority of FAERS reports, therefore, originate from the United States, although international pharmacovigilance reporting represents a substantial component of the database.

Product-level distribution of adverse event reports

The AEMS dashboard does not provide a native interface for ranking products by total adverse event report counts. To address this limitation, product-level aggregation was performed using the OpenFDA API [8].

Analysis of FAERS data identified several products with the highest number of reported adverse events. The most frequently reported products included adalimumab (Humira), etanercept (Enbrel), dupilumab (Dupixent), ranitidine (Zantac), and lenalidomide (Revlimid), followed by commonly used medications such as aspirin, metformin, prednisone, amlodipine, and methotrexate. These findings reflect products with high reporting volumes in FAERS; however, report counts are influenced by factors such as prescribing frequency, duration of market availability, and reporting practices and should not be interpreted as direct measures of drug risk.

A summary of the most frequently reported products is presented in Table 4.

**Table 4:** Top 10 products by adverse event report count in FAERS via OpenFDA analysis Top 10 products ranked by total number of adverse event reports identified using the OpenFDA API based on FAERS data available at the time of analysis (March 11, 2026). Product counts reflect aggregation by medicinal product name using exact string matching and may include duplicate entries corresponding to the same active ingredient due to variability in naming conventions. Report counts represent reporting frequency and should not be interpreted as measures of incidence, causality, or comparative safety risk. FAERS: Food and Drug Administration Adverse Event Reporting System; API: application programming interface

Rank	Product	Total reports
1	Humira	662,612
2	Enbrel	573,237
3	Dupixent	399,921
4	Zantac	356,186
5	Revlimid	350,843
6	Aspirin	492,061
7	Metformin	289,723
8	Prednisone	482,820
9	Amlodipine	257,744
10	Methotrexate	403,320

## Discussion

Unlike earlier FAERS access methods that relied on downloading and processing periodic quarterly data releases, the AEMS dashboard provides a near-real-time, integrated visualization platform that enables direct exploratory analysis of adverse event data without the need for external data infrastructure. This distinction represents a meaningful shift from FAERS as a raw data repository to AEMS as an accessible analytic interface, potentially facilitating exploratory pharmacovigilance analyses and lowering technical barriers for clinicians and researchers.

This study provides a descriptive overview of adverse event reports submitted to the FAERS, highlighting patterns in reporting across time and patient demographics. The findings demonstrate continued growth in adverse event reporting within the FAERS database and reveal notable demographic differences in reporting patterns.

One of the key observations from this analysis is the predominance of reports among female patients. Sex-based differences in adverse drug reactions have been documented in previous pharmacovigilance research and may reflect a combination of biological, pharmacokinetic, and behavioral factors [9]. Women often exhibit differences in drug metabolism, hormone-related pharmacodynamics, and body composition that can influence drug response and susceptibility to adverse effects [10]. Additionally, women generally have higher healthcare utilization rates and may be more likely to report medication-related concerns, which could contribute to higher representation in spontaneous reporting systems [11].

The data also demonstrate a steady increase in adverse event reporting over time. This trend likely reflects several factors, including expanded drug utilization, improvements in pharmacovigilance infrastructure, increased regulatory requirements for manufacturer reporting, and greater awareness of medication safety among healthcare professionals and patients [2]. The growing accessibility of electronic reporting systems may also contribute to increased submission of safety reports. Importantly, increases in reporting volume should not be interpreted as a direct indication of rising incidence of adverse drug reactions but rather as an indicator of enhanced surveillance and reporting activity.

Spontaneous reporting systems such as FAERS play a crucial role in post-marketing drug safety monitoring [2]. While pre-approval clinical trials provide valuable information about drug efficacy and common adverse events, they are typically conducted in relatively controlled environments with limited sample sizes and strict eligibility criteria. As a result, rare adverse events, long-term safety outcomes, and adverse reactions occurring in broader patient populations may only become apparent after widespread clinical use. Pharmacovigilance databases, therefore, serve as an important mechanism for identifying potential safety signals that may warrant further investigation.

Despite its value for drug safety monitoring, FAERS data have several well-recognized limitations. Reporting to FAERS is largely voluntary for healthcare professionals and consumers, which introduces the potential for both underreporting and reporting bias [12]. Certain adverse events may be more likely to be reported due to media attention, regulatory warnings, or litigation, while other events may remain unreported. In addition, individual reports may contain incomplete or missing information regarding patient characteristics, drug exposure, comorbidities, or treatment duration, which limits the ability to perform detailed clinical analyses.

Another important limitation is that FAERS lacks reliable data on the total number of individuals exposed to specific medications. Without information on drug exposure or population denominators, it is not possible to calculate incidence rates or directly compare the risk of adverse events across different drugs [13]. Furthermore, many reports involve multiple concomitant medications, making it difficult to determine which product may have contributed to the reported event.

Because of these limitations, findings derived from FAERS analyses should be interpreted as potential safety signals rather than definitive evidence of causal relationships between medications and adverse events [14]. Signals identified through spontaneous reporting systems should ideally be evaluated through additional epidemiologic studies, clinical investigations, or other pharmacovigilance methodologies to better assess potential drug-related risks.

Another limitation of the AEMS interface is the absence of native functionality to rank products by total adverse event report volume. While the dashboard supports efficient exploration of adverse events across multiple dimensions, key aggregate queries, such as identification of the most frequently reported products, require external data processing. This reflects the early developmental stage of the platform and underscores the need for complementary analytic methods for more advanced pharmacovigilance use cases. Future enhancements that incorporate native product-level ranking and expanded query capabilities would substantially strengthen the utility of AEMS for both exploratory and applied pharmacovigilance research.

Future research may benefit from integrating FAERS data with additional real-world data sources such as electronic health records, insurance claims databases, or clinical registries. Combining multiple data sources may help improve signal validation, strengthen causal inference, and provide a more comprehensive understanding of medication safety in real-world clinical practice.

The AEMS platform also reflects a broader integration of adverse event reporting systems across FDA-regulated products [6], with plans to incorporate data from additional surveillance systems such as the Vaccine Adverse Event Reporting System (VAERS) [15] and the Manufacturer and User Facility Device Experience (MAUDE) database [16], as well as other legacy reporting systems. This broader rollout was also described by the FDA at launch.

Overall, the present analysis underscores the continued importance of pharmacovigilance systems such as FAERS in supporting post-marketing drug safety surveillance and identifying potential safety signals that may inform regulatory decision-making and clinical practice.

## Conclusions

The newly released FDA AEMS dashboard represents a significant advancement in public access to pharmacovigilance data by providing an interactive platform for exploring FAERS reports without requiring specialized data processing. Early evaluation demonstrates that the system enables rapid descriptive analysis of large-scale adverse event datasets. While the dashboard improves accessibility and transparency, interpretation of FAERS data must remain cautious due to the inherent limitations of spontaneous reporting systems. Future research may leverage the AEMS platform to identify emerging safety signals and support post-marketing drug safety monitoring.
